# Absence of *Cryptosporidium hominis* and dominance of zoonotic *Cryptosporidium* species in patients after Covid-19 restrictions in Auckland, New Zealand

**DOI:** 10.1017/S0031182021000974

**Published:** 2021-09

**Authors:** M. A. Knox, J. C. Garcia-R, P. Ogbuigwe, A. Pita, N. Velathanthiri, D. T. S. Hayman

**Affiliations:** Massey University, School of Veterinary Science, Palmerston North, Manawatu-Wanganui, New Zealand

**Keywords:** Covid-19, *Cryptosporidium*, New Zealand, nonpharmaceutical intervention, public health

## Abstract

Coronavirus disease-2019 (Covid-19) nonpharmaceutical interventions have proven effective control measures for a range of respiratory illnesses throughout the world. These measures, which include isolation, stringent border controls, physical distancing and improved hygiene also have effects on other human pathogens, including parasitic enteric diseases such as cryptosporidiosis. *Cryptosporidium* infections in humans are almost entirely caused by two species: *C. hominis,* which is primarily transmitted from human to human, and *Cryptosporidium parvum,* which is mainly zoonotic. By monitoring *Cryptosporidium* species and subtype families in human cases of cryptosporidiosis before and after the introduction of Covid-19 control measures in New Zealand, we found *C. hominis* was completely absent after the first months of 2020 and has remained so until the beginning of 2021. Nevertheless, *C. parvum* has followed its typical transmission pattern and continues to be widely reported. We conclude that ~7 weeks of isolation during level 3 and 4 lockdown period interrupted the human to human transmission of *C. hominis* leaving only the primarily zoonotic transmission pathway used by *C. parvum.* Secondary anthroponotic transmission of *C. parvum* remains possible among close contacts of zoonotic cases. Ongoing 14-day quarantine measures for new arrivals to New Zealand have likely suppressed new incursions of *C. hominis* from overseas. Our findings suggest that *C. hominis* may be controlled or even eradicated through nonpharmaceutical interventions.

## Graphical abstract

**Figure:**
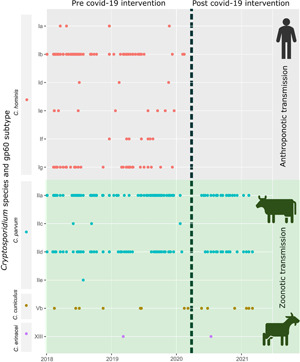

## Introduction

Coronavirus disease-2019 (Covid-19) was declared a global pandemic by the World Health Organization (WHO) on 11 March 2020, leading to a variety of responses by different governments around the world. The nonpharmaceutical intervention strategy of New Zealand was particularly stringent and effectively eliminated a burgeoning Covid-19 outbreak through a nationwide lockdown, physical distancing, improved hand hygiene and ongoing 14-day quarantine for returning travellers (Robert, 2020; Baker *et al*., 2020*a*). A four-level alert system (New Zealand Government, 2020) was introduced on 21 March, beginning at level 2 and quickly moving to level 3 on 23 March and level 4 (i.e. the entire nation goes into self-isolation) on 25 March. The level 4 lockdown remained in place until 27 April, when it was reduced to level 3. By 11 May it was lowered to level 2 and finally decreased to level 1 on 8 June (Baker *et al*., 2020*b*). The primary intention of these measures in New Zealand was to reduce the transmission of Covid-19, but they also brought about a reduction in influenza and other respiratory viral infections in 2020 (Huang *et al*., 2021). Similar impacts have also been observed elsewhere (Fricke *et al*., 2021; Yang *et al*., 2021), but little attention to date has been given to parasitic enteric diseases.

*Cryptosporidium* species are obligatory intracellular intestinal parasites that infect a wide range of vertebrate hosts, causing a considerable burden of gastrointestinal disease (Xiao *et al*., 2004; Garcia and Hayman, 2016). In New Zealand, cryptosporidiosis is a notifiable disease with a higher incidence compared to other developed countries (Learmonth *et al*., 2004). Two species, *C. hominis* and *C. parvum* are responsible for the majority of human infections globally, and in New Zealand (Garcia-R and Hayman, 2017; Garcia-R *et al*., 2017; Garcia-R *et al*., 2020*b*). Of these, *C. hominis* is transmitted primarily through human-to-human contact whereas *C. parvum* is mainly zoonotic with ruminants as the primary reservoir host [(Learmonth *et al*., 2004; Feltus *et al*., 2006; Leitch and He, 2011) though see King *et al*. (2019)]. Clear seasonal peaks in cryptosporidiosis are present in New Zealand. An early autumnal peak for *C. hominis* associated with increased recreational water use and a spring peak for *C. parvum* associated with dairy cattle calving season is observed each year (Learmonth *et al*., 2004; Lake *et al*., 2008; Garcia-R *et al*., 2020*b*). Transmission of both species is through the faecal−oral route, following contact with infected hosts or water contaminated with the transmissive stages (oocysts) of the organism (Bouzid *et al*., 2013).

One of the difficulties with monitoring cryptosporidiosis is the lack of morphological characters to identify different *Cryptosporidium* species. Frequently, diagnosis is based on microscopy by the presence of oocysts in stool samples of infected patients or enzyme immunoassay and species-level identification is rarely attempted. Reliable species and subtype level identification is possible through molecular methods, though diagnostic laboratories moving to molecular diagnostics typically utilize qPCR and report presence/absence, without speciation. The most widely used marker for this purpose is the 60 kDa glycoprotein locus (gp60), upon which the subtyping of *Cryptosporidium* species is based (Feng and Xiao, 2017). This classification scheme has been used to study the epidemiology of human outbreaks of cryptosporidiosis in many previous studies (O'Brien *et al*., 2008; Chalmers *et al*., 2019). Here, we examine the disease dynamics of *Cryptosporidium* infections using gp60 for species identification and subtype family diversity patterns before, during and after lockdown measures in New Zealand.

## Materials and methods

Anonymous *Cryptosporidium*-positive stool samples were collected between 2015 and 2021 through routine public health investigation activities from symptomatic humans visiting private and public general practitioner (GP) surgeries and hospitals throughout New Zealand. Samples were sent from accredited diagnostic laboratories to the Protozoa Research Unit at Hopkirk Research Institute (Massey University) for molecular analyses under a Ministry of Health contract. Stools were collected in 10-ml screw-cap tubes and stored at 4°C in the laboratory until DNA extraction.

DNA extraction was carried out 1–2 weeks after receiving samples at the Hopkirk Research Institute. Before DNA extraction, the oocysts of *Cryptosporidium* were physically disrupted using a beadbeater (Tissue Lyser II, Qiagen) at 30 Hz for 5 min. DNA extractions were performed using Zymo Quick-DNA Fecal/Soil Microbe kits following the manufacturer's instructions. A fragment of the gp60 gene was amplified using a nested PCR (Alves *et al*., 2003; Waldron *et al*., 2009) and amplification products were sequenced in both directions using Big Dye Terminator version 3.1 reagents on an ABI 3730XL automated DNA sequencer (Applied Biosystems, Foster City, California, USA).

*Cryptosporidium* gp60 sequences were classified into species and subtype families according to guidelines in Feng and Xiao (2017). While our sampling prior to 2020 included a wide range of regions throughout New Zealand, the 2020 samples were dominated by the Auckland region (118/130). For this reason, we filtered out results from elsewhere and limited our analyses to samples collected from the Auckland region. To check the representativeness of our dataset, we compared it with data from the national notifiable disease surveillance website (https://surv.esr.cri.nz/surveillance/annual_diseasetables.php).

## Results

**Fig. 1.**
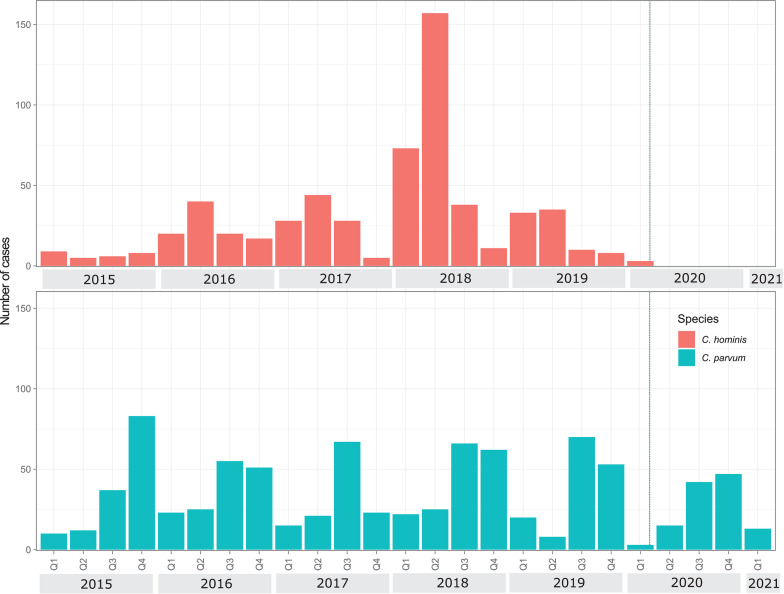
*Cryptosporidium hominis* and *C. parvum* quarterly cases from Auckland, New Zealand 2015–2021. Dotted line in Q1 2020 indicates the start of Covid-19 national lockdown.

Our dataset included 1502 sequences received from 9 January 2015 to 3 March 2021 (Supplementary Table 1). Of these, *C. parvum* was the most common (*n* = 868), followed by *C. hominis* (598), *Cryptosporidium cuniculus* (28) and *Cryptosporidium erinacei* (8). Seasonal dynamics of *C. hominis* and *C. parvum* before 2020 and the impact of the Covid-19 lockdown are illustrated in Fig. 1. *Cryptosporidium hominis* was commonly found in the first (Q1) and second quarters (Q2) of the year between January and June while *C. parvum* was more common in the third (Q3) and fourth quarters (Q4) from July to December. The exception to this is in 2020, when no *C. hominis* was detected after 14 February 2020 (Fig. 1).

**Fig. 2.**
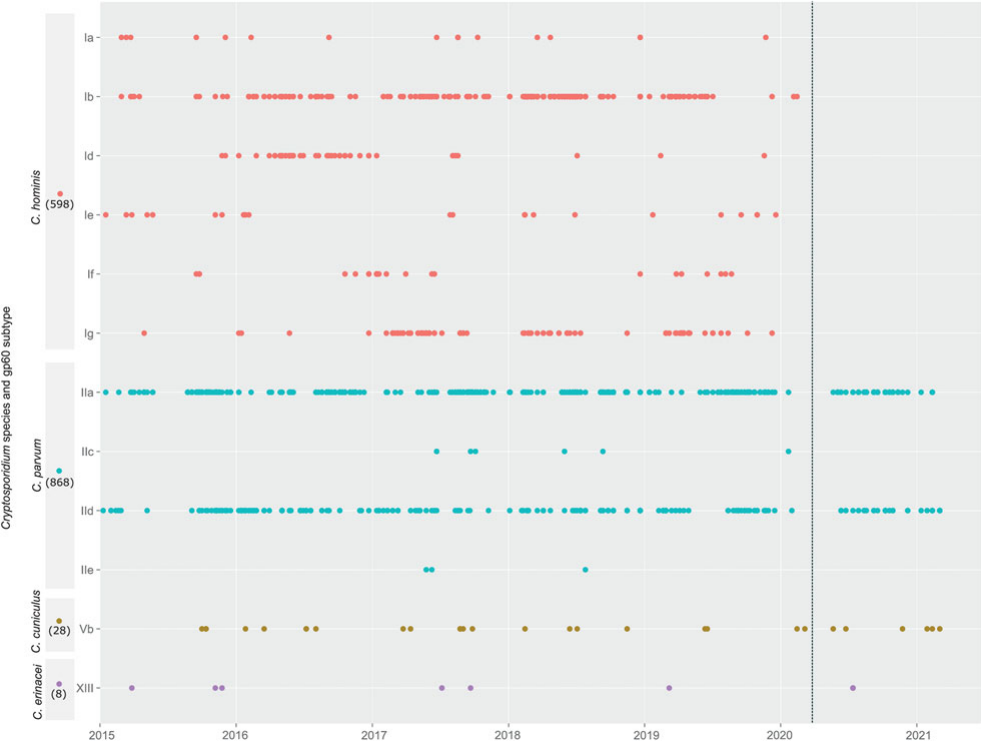
Timeline of *Cryptosporidium* gp60 subtype cases identified from Auckland, New Zealand 2015–2021. Number of cases per species are indicated on the *y* axis. Dotted line in 2020 indicates the start of Covid-19 national lockdown.

Further examination of gp60 subtype families shows the diversity and temporal dynamics of *C. hominis* and *C. parvum* (Fig. 2). Within *C. hominis*, subtype family Ib was detected consistently between 2015 and the beginning of 2020. Other gp60 subtype families in *C. hominis* appeared as sporadic outbreaks e.g. Id in 2016, Ig in 2017, 2018 and 2019 and/or single cases e.g. Ia in 2015–2019. Within *C. parvum,* subtype families IIa and IId were consistently detected whilst IIc and IIe appeared as sporadic single cases. Of the other zoonotic *Cryptosporidium* species, *C. cuniculus* and *C. erinacei* were only detected occasionally.

Our data are representative of the total number of cases reported nationally from the notifiable disease database from 2015 to 2019 (between 33% and 46%, Supplementary Table 1). However, in 2020 the number of cases reported to our lab dropped to a lower proportion (18%). This was probably caused by extra stress on diagnostic laboratories that did not send as many samples to our lab for molecular analyses during and following the Covid-19 outbreak. Data from outside Auckland are not included in our analyses due to low numbers in 2020, but in the few samples from outside Auckland which we do have, no post lockdown cases of *C. hominis* have been reported.

## Discussion

In response to Covid-19, New Zealand underwent a nationwide lockdown beginning in late March 2020, with households spending over 7 weeks at home except for essential personal movements like supermarket and hospital visits. Since this time, our surveillance has revealed that *C. hominis* has not been detected in reported cases of cryptosporidiosis from the Auckland region. This absence has continued until the time of publication during the first quarter of 2021, which in previous years has been the beginning of peak season for *C. hominis* infections. In contrast, *C. parvum* and other less common zoonotic *Cryptosporidium* species have continued typical seasonal patterns. Our data suggest that the national measures for responding to the Covid-19 also interrupted *C. hominis* transmission. For instance, the closure of all child care centres and schools may have limited *C. hominis* transmission since these have been linked to previous outbreaks (Goñi *et al*., 2015). Ongoing managed isolation of visitors or citizens returning to New Zealand since then has also likely reduced new imported cases of cryptosporidiosis.

Prior to 2020, infections by *C. hominis* were reported seasonally (Garcia-R and Hayman, 2017; Garcia-R *et al*., 2020*b*). Furthermore, temporal dynamics of gp60 subtype families generally revealed sporadic infection patterns. Subtype families Ia, Id, Ie, If and Ig appeared at irregular intervals throughout the study period, which may indicate an importation by visitors or returning travellers from a global pool of *C. hominis* that fail to establish within New Zealand or an opportunistic behaviour of the parasite (Garcia-R and Hayman, 2017; Garcia-R *et al*., 2020*a*). The majority of *C. hominis* cases was subtype family Ib, which is also the most common subtype family globally (Feng *et al*., 2009; Putignani and Menichella, 2010; Segura *et al*., 2015). These findings imply that despite the hardiness of *Cryptosporidium* oocysts in the environment (Carey *et al*., 2004), historical *C. hominis* infections in New Zealand arise through importation and that the levels of sanitation and hand hygiene, as well as a low-density population, may be sufficient to prevent the establishment of endemic *C. hominis* infections. Similarly, improved hand hygiene on farms, especially during calving season may help control zoonotic *C. parvum* transmission in New Zealand (Thomas-Lopez *et al*., 2020).

*C. parvum* sequences were dominated by gp60 subtype families IIa and IId, which both occurred consistently throughout the study period, including during 2020. These *C. parvum* subtype families are primarily associated with ruminants and transmitted through them to humans (Mammeri *et al*., 2019; Nader *et al*., 2019). Both have been detected in cattle and sheep in New Zealand (Garcia-R *et al*., 2017) and evidence from overseas suggests that subtype family IId may be more prevalent in sheep and goat populations (Quílez *et al*., 2008), but further research is needed to identify if host-specific *C. parvum* subtype families occur in New Zealand's domestic animals. Overall, *C. parvum* transmission was not interrupted by Covid-19 control measures except perhaps by a small reduction in peak numbers during 2020 Q3 and Q4. Subtype family IIc is rare in New Zealand, with only six cases in our dataset. Previous research shows that unlike other *C. parvum* subtype families, IIc is an anthroponotic (human-adapted) and a potential emerging genotype (King *et al*., 2019; Nader *et al*., 2019). While no cases of subtype family IIc were detected after Covid-19 control measures, its rarity before Covid-19 prevents any strong conclusions from our data. Further investigation of gp60 diversity, i.e. trinucleotide repeat regions, may have revealed more details of the transmission patterns of cryptosporidiosis in New Zealand, but this would have been difficult to assess since we do not have descriptive epidemiology or exposure data for our cases. In *C. parvum*, previous research has demonstrated strong associations between animal contact and cryptosporidiosis, though human-to-human transmission is also possible (Chalmers *et al*., 2019). However, like *C. hominis*, we suggest that human to human *C. parvum* outbreaks in New Zealand are contained through basic sanitation measures.

Our study has limitations in the methodology of data collection stemming from significant changes to human behaviours during 2020 which may have influenced our results. The number of samples we have for 2020/2021 is lower than in previous years and adds uncertainty in the data. As well as potentially being due to a reduction in transmission and cases, since the symptoms of cryptosporidiosis do not closely match Covid-19, people with cryptosporidiosis may have been less likely to visit doctors immediately prior to, during and following Covid-19 restrictions compared with previous years. Furthermore, because symptoms usually resolve themselves without treatment, cryptosporidiosis is an under-reported disease throughout the developed world (Dreesman *et al*., 2007; Scallan *et al*., 2011). Despite being a notifiable disease in New Zealand it is likely that only a small proportion of cases are diagnosed, thus raising the potential for bias in reporting different *Cryptosporidium* species. The severity of cryptosporidiosis is impacted by many factors including host (concurrent infection, malnutrition, immunosuppression, age), environmental (dose of exposure) as well as species and genotype of *Cryptosporidium* (Weir, 2001). Of the species in our study, *C. hominis* is associated with more severe infections and higher shedding rates (Bushen *et al*., 2007), potentially making reporting and detection of *C. hominis* more likely than *C. parvum*. Once cryptosporidiosis cases are confirmed, the diagnostic laboratories send stool samples to our lab for genetic analyses, but this may not be nationally representative as evidenced by the lack of samples from outside the Auckland region during 2020. The reasons for this are unclear but may reflect a focus on essential services and over-stressed health systems not having time to send samples since the start of Covid-19.

In conclusion, our study revealed that nonpharmaceutical interventions greatly reduced *C. hominis* in Auckland, New Zealand. Since the March 2020 national lockdown, travel restrictions and mandatory visitor isolation have kept reported *C. hominis* numbers at zero. Ongoing monitoring of *Cryptosporidium* species and gp60 subtype families as international travel restrictions ease will provide further information on cryptosporidiosis in New Zealand. Finally, to further investigate the preliminary findings presented here we recommend future studies focus on descriptive epidemiology and exposure data for cases of human cryptosporidiosis in New Zealand.

## Data Availability

Sequence data availability is outlined in Garcia-R et al. (2020*b*).

## Supplementary material

For supplementary material accompanying this paper visit https://doi.org/10.1017/S0031182021000974. click here to view supplementary material
